# A model of hippocampal cell assembly dynamics based on single-cell theta phase precession

**DOI:** 10.1186/1471-2202-14-S1-P402

**Published:** 2013-07-08

**Authors:** Angus Chadwick, Matthew F Nolan, Mark CW Van Rossum

**Affiliations:** 1 Institute for Adaptive and Neural Computation, University of Edinburgh, EH8 9AB, UK; 2 Neuroinformatics Doctoral Training Centre, University of Edinburgh, EH8 9AB, UK; 3 Centre for Integrative Physiology, University of Edinburgh, EH8 9XD, UK

## 

Neural oscillations are associated with a wide variety of cognitive and perceptual processes, both in health and disease. In the hippocampus of rodents during exploratory behaviours, prominent theta and gamma oscillations are observed in the local field potential (LFP). These rhythms are linked to spatial cognition and working memory, but the underlying mechanisms are unclear. At the single-cell and cell assembly levels, two salient features emerge during the theta rhythm - phase precession and spatiotemporal spike sequences. The relationship between these two phenomena is yet to be fully characterised. For example, it is unclear whether the sequential structure of hippocampal cell assemblies is fully explained through independent phase coding at the single-cell level, or whether further coordination is required to account for the observed multi-cell behaviour.

We developed a descriptive model of phase coding in individual place cells and used this model to investigate the cell assembly dynamics on a linear track. Under the assumption of independent phase coding, key experimental quantities were derived analytically and their relationship to behavioural variables was analysed and compared to experimental data (e.g., [1]). We showed that experimentally established relationships between behavioural variables such as running speed and cell assembly metrics such as the compression factor and lookahead can be reproduced and understood analytically in terms of the collective behaviour of independent phase coding units.
